# Screening of Microbes Associated With Swine Growth and Fat Deposition Traits Across the Intestinal Tract

**DOI:** 10.3389/fmicb.2020.586776

**Published:** 2020-10-16

**Authors:** Shi Tang, Ying Xin, Yunlong Ma, Xuewen Xu, Shuhong Zhao, Jianhua Cao

**Affiliations:** ^1^College of Animal Science and Technology, Huazhong Agricultural University, Wuhan, China; ^2^The Cooperative Innovation Center for Sustainable Pig Production – Swine Breeding and Reproduction Innovation Platform, Huazhong Agricultural University, Wuhan, China

**Keywords:** pig, 16S RNA, microbiota, intestinal gut segment, meat qualities

## Abstract

Pigs, as one of the most common livestock species worldwide, are expected to have a fast growth rate and lower subcutaneous fatness but higher intramuscular fat (“marbling meat”). Nowadays, it is believed that not only host genetics but also its gut microbiomes can modulate farm animal phenotypes, however, many of the mechanisms remain elusive. We measured the body weight (BW), average daily gain (ADG), backfat thickness (BFT), and intramuscular fatness (IMF) of 91 Enshi pigs at 260 days of age, then genotyped each one individually using a 50K single nucleotide polymorphism array and performed 16S ribosomal RNA gene sequencing on 455 microbial samples from the jejunum, ileum, cecum, colon, and rectum. The microbial diversity showed notable spatial variation across the entire intestinal tract, with the cecum and colon having the highest α-diversity. The cecal and colonic microbiotas made greater contributions to BW and ADG and accounted for 22–37% of the phenotypic variance. The jejunal and cecal microbiotas contributed more (13–31%) to the BFT and IMF than the other segments. Finally, from cecum, colon, and jejunum, we identified eight microbial taxa that were significantly correlated with the target traits. The genera *Alloprevotella* and *Ruminococcaceae* UCG-005 were highly positively correlated with BW and ADG. The genera *Prevotellaceae* UCG-001 and *Alistipes* in the cecum and *Clostridium sensu stricto 1* in the jejunum were highly positively correlated with BFT and IMF. The genera *Stenotrophomonas*, *Sphaerochaeta*, and *Desulfovibrio* were negatively associated with the mentioned traits. These findings could aid in developing strategies for manipulating the gut microbiota to alter production performance in pigs.

## Introduction

Pigs are one of the most important livestock species that produce meat and supply proteins for human consumption worldwide (Boland et al., 2013). Geneticists and breeders expect to cultivate pig breeds with rapid growth rate, less backfat, and more intramuscular fat (“marbling meat”), which are traits that substantially affect the economic values of pigs as livestock. These complex traits are thought to be determined by genetic variants and are influenced by external factors such as nutritional adequacy, health states, and environmental conditions (Newton-Cheh and Hirschhorn, 2005). Many scientists are now focused on the gut microbiota, and its composition in the gut is a key part of all mammals. Accumulating evidence from humans, mice, and farm animals suggests a strong link between gut microbes and complex traits such as adiposity (Bäckhed et al., 2004; Turnbaugh et al., 2006, Turnbaugh et al., 2009;,  Camarinha-Silva et al. 2017). One study showed that germ-free mice colonized with “microbiotas” from obese mice exhibited much more body fat than mice colonized with “microbiotas” from lean ones (Turnbaugh et al., 2006), thus lending credibility to the role of the gut microbiota in adiposity.

The intestinal microbiota provides many extra functions for pigs, such as enhanced energy-harvesting capacity, short-chain fatty acid (SCFA) production by anaerobic fermentation, and improved resistance to pathogenic bacteria (Lamendella et al., 2011). Hence, gut microbes should be considered as novel and steady factors that influence animal complex traits. Consistently, pig feed efficiency (Yang et al., 2017) and fatness traits (Fang et al., 2017) are associated with the gut microbiota, possibly by fermenting dietary polysaccharides, which leads to excessive SCFA production in the gut. However, many of these traits–microbiota association studies mainly focused on the fecal microbiota or microbes in one specific gut segment such as the cecum (Holman et al., 2017). The pig’s gastrointestinal tract consists of several segments with distinctive appearances and physical structures, including the duodenum, jejunum, ileum, cecum, colon, and rectum. Holman et al. (2017) performed a meta-analysis with 20 published datasets and found that the swine gut microbial structure and composition differed significantly among gastrointestinal tract sections. The enterotypes, which are classifications of living organisms based on their bacteriological ecosystem, also differ significantly between gut sections in farm animals (Yuan et al., 2020). Yan et al. (2019) suggested that the fecal microbial community should be used cautiously as a proxy of the gut microbiota in association studies between gut microbes and animal target traits.

Hence, for different traits, the degree of importance of each gut segment should be evaluated. The term “microbiability” (*m*^2^), proposed by Difford et al. (2018), is the proportion of total variance explained by the microbiome for target traits and is the appropriate index for evaluating the importance of each gut section. Wen et al. (2019) estimated the *m*^2^ of each gut section on abdominal fat weight in chickens and found that the duodenum (0.24) and cecum (0.21) had much higher *m*^2^ values than the other gut segments (0.02–0.06). These researchers considered the duodenum and cecum to be segments that should be further screened for causal microbes involved in fat metabolism (Wen et al., 2019). However, to our knowledge, most previous studies on pigs did not apply this strategy.

Therefore, in the current study, we genotyped 91 pigs using a 50K single nucleotide polymorphism (SNP) array and performed 16S ribosomal RNA (rRNA) sequencing on 455 samples from five gut segments (jejunum, ileum, cecum, colon, and rectum) to comprehensively investigate the contribution of the gut microbiota to swine economic traits [i.e., body weight (BW), average daily gain (ADG), backfat thickness (BFT), and intramuscular fat (IMF)] and to further identify the causal microbes of these targets traits and reveal their interaction with host genetics.

## Materials and Methods

### Sample Collection and Phenotype Determination

A total of 91 Chinese indigenous pigs (Enshi black pigs; 50 castrated boars and 41 sows) were tested in this study. All pigs were raised on the same farm under standardized conditions, with free access to water and a diet based on corn and soybean meal. Male pigs were castrated at 10 days old. All pigs were healthy and had not been treated with antibiotics for at least 3 months before sampling. The pigs were slaughtered at 260 ± 5 days of age, and each pig’s gross weight was measured using a scale with an accuracy of 0.1 kg. BFTs at the shoulder, chest, and waist were measured using a Vernier caliper (Mitutoyo 102-3, Japan), and the average BFT was calculated. Intestinal contents were sampled from the jejunum, ileum, cecum, colon, and rectum, snap-frozen in liquid nitrogen, and stored at −80°C until sequencing. Blood samples were obtained from the ear and stored at −20°C. Longissimus dorsi samples were obtained to measure the IMF. All meat samples were oven-dried to a constant weight, then ground into powder. The routine Soxhlet extraction method was used to extract the fat, and the IMF was calculated. The Animal Care and Use Committee of Huazhong Agricultural University approved this study.

### Host Genotyping and Quality Control

Pig DNA was extracted from ear blood samples using the routine phenol/chloroform extraction method. All pigs were genotyped using Illumina Porcine 60K SNP Chips (Illumina, San Diego, CA, United States) per the manufacturer’s protocol. The software of PLINK (v.1.90) was used for quality control with the filtering criteria: sample call rate > 90%, SNP call rate > 95%, minor allele frequency > 1%, and Hardy-Weinberg equilibrium *p*-value < 10^–6^. In total, 51,315 SNPs were filtered and retained for subsequent analysis.

### 16S Ribosomal RNA Sequencing and Analysis

The gut lumen samples were thawed and homogenized separately, and ∼200 mg of each sample was used to extract the microbial genomic DNA using the QIAamp DNA Stool Mini Kit (Qiagen, Hilden, Germany). DNA concentrations were monitored via 1% agarose gels, then diluted to 1 ng/μl using distilled water. The V4 region of the 16S rRNA gene was amplified with the primer pair 515F/806R. All PCR reactions were carried out in 30-μl solutions with 0.2 μM of primers, ∼10-ng template DNA, and 15 μl of Phusion High-Fidelity PCR Master Mix (New England Biolabs). The Ion Plus Fragment Library Kit (Thermo Scientific) was used to construct the sequencing libraries, which were sequenced on the Ion S5TM XL platform to generate 300-bp single-end reads.

The raw reads were quality filtered as per Wen et al. (2019). All clean reads were aligned and clustered into features [or operational taxonomic units (OTUs)] at a 100% similarity threshold by DATA2 that embedded in QIIME2 protocol (Bolyen et al., 2019). OTUs with relative abundances < 10^–6^ or appeared in < 3 pigs were omitted, then the OTU abundances were calculated. Taxonomies were assigned to obtain the taxonomic classification of each OTU from phylum to species using the reference database, SILVA release 132 (Quast et al., 2012).

The α-diversity (Shannon index, observed OTUs, and Faith’s phylogenetic diversity) and β-diversity (Bray–Curtis matrix) parameters were calculated from qualified OTU data with algorithms embedded in QIIME2 (Bolyen et al., 2019). The principal coordinate analysis was performed with the Bray–Curtis matrix using the ape package in R (Paradis et al., 2004). Tax4fun2 (Wemheuer et al., 2018) was used to predict the potential functions of the microbiotas identified in the jejunum, ileum, cecum, colon, and rectum.

### Single Nucleotide Polymorphism-Based Heritability Estimation

The 51,315 filtered SNPs were used to estimate the principal components, genetic relatedness matrix (GRM), and the SNP-based heritability using Genome-Wide Complex Trait Analysis (GCTA) software (ver 1.93.1; Yang et al., 2011). The GRM estimation model used was:

$$g_{i\,j}=\frac{1}{N}\,\sum_{v=1}^{N}\frac{\left(x_{i\,v}-2\,\bar{p_{v}}\right)\,\left(x_{j\,v}-2\,\bar{p_{v}}\right)}{2\,\bar{p_{v}}\,\left(1-\bar{p_{v}}\right)}$$

in which *g*_*ij*_ is the genetic similarity between pigs *i* and *j*; *x*_*iv*_ and *x*_*jv*_ represent the number of reference alleles in pigs *i* and *j*; $\bar{p_{v}}$ is the reference allele frequency; and *N* is the SNP number.

The SNP-based heritability of the target traits was estimated with the model:

y=K⁢c+g+e    [A]

where *y* is a vector of the phenotype; *c* is a vector of fixed covariates, including sex effect and the first five host genetic principal components; *K* is the corresponding matrix for *c*; and g is a vector of the total effects of all SNPs with $∼N(0,G\sigma_{A}^{2}$), where *G* and $\sigma_{A}^{2}$ are the GRM and genetic variance, respectively; and e is the residual effect.

### Operational Taxonomic Unit Abundance-Based Microbiability Estimation

To estimate the microbiability, the microbial relationship matrix (MRM) was used to construct all OTUs from each normalized segment. The MRM was constructed from the following equation described in Wen et al. (2019):

$$m_{\mathrm{sij}}=\frac{1}{N}\,\sum_{o=1}^{N_{s}}\frac{\left(x_{\mathrm{sio}}-\bar{x_{\mathrm{so}}}\right)\,\left(x_{\mathrm{sjo}}-\bar{x_{\mathrm{so}}}\right)}{\sigma_{\mathrm{so}}^{2}}$$

where *m*_sij_ is the microbial similarity in segment *s* between pigs *i* and *j*; *x*_sio_ and *x*_sjo_ are the respective counts of OTU *o* in segments *s* in pigs *i* and *j*; $\bar{x_{\mathrm{so}}}$ is the average count of OTU *o* in segment *s* for the whole population; $\sigma_{\mathrm{so}}^{2}$ is the count variance of OTU *o* in segment *s*; and *N*_*s*_ is the total OTU count in segment *s*.

The microbiability, or the fraction of the gut microbial variance to phenotypic variance, was calculated with the following model:

$$y=K\,c+m_{s}+e\;\left[B\right]$$

where *y*, *c*, *K*, and *e* are the same as in model [A]. *m*_*s*_ is the gut microbiota effect in segment *s* following the multinomial distribution $m_{s}∼N(0,M\sigma_{m}^{2}$). The microbiability was estimated with GCTA software using the MRM instead of the GRM.

### Identification of Candidate Microbes for Target Traits

All individuals were sorted by their trait phenotypes (BW, ADG, BFT, and IMF). The top and bottom 20% of the pigs (*N* = 18) were deemed as two disparate groups, and each two-group pair was statistically compared using the Wilcoxon rank-sum test for the microbes constituting > 70% of the top and bottom groups. Spearman’s and Pearson’s correlations were calculated among the screened candidate microbes and target traits using the psych package in R.

## Results

### Descriptive Statistics for Host Phenotypes

Table 1 lists the descriptive statistics for the host traits. The pig breed used in the current study was characterized by small size (81.12 kg at 260 days), low growth rate (mean, 310 g/day), excellent meat quality (3.6% IMF), and strong fat accumulation ability (36.53 mm BFT). Additionally, this pig population, as a Chinese local breed, has not been strongly selected for any traits. Subsequently, all measured traits displayed high variable coefficients (22.16-48.16%), indicating that the genetic background and intestinal microbial status of this population were in a primitive state without manual intervention and were suitable for analyzing the effects of host genetics and gut microbes on complex traits.

**TABLE 1:** Descriptive statistics for host phenotypes.

**Traits**	**Sex**	***N***	***Mean***	***SD***	**CV (%)**	**Max**	**Min**
BW (kg)	♂	51	81.41	19.77	24.29	141.50	34.40
	♀	40	80.40	15.93	19.81	118.50	47.00
	total	91	81.12	18.00	22.16	141.5	34.4
ADG (g)	♂	51	314.46	83.38	26.52	537.02	127.27
	♀	40	304.30	61.66	20.26	448.47	181.50
	total	91	310.28	74.97	24.26	537.02	127.27
ABFT (mm)	♂	51	37.15	10.44	28.11	61.73	16.04
	♀	40	35.74	8.90	24.90	58.71	19.16
	total	91	36.53	9.65	26.42	61.73	16.04
IMF (%)	♂	51	3.41	1.87	54.74	8.64	1.25
	♀	40	3.05	1.08	35.48	5.24	1.23
	total	91	3.26	1.57	48.16	8.64	1.23

*ABTF, average backfat thickness. These two symbols mean male and female respectively.*

### Microbial Compositions and Functions in Swine Gut Segments

**FIGURE 1:**
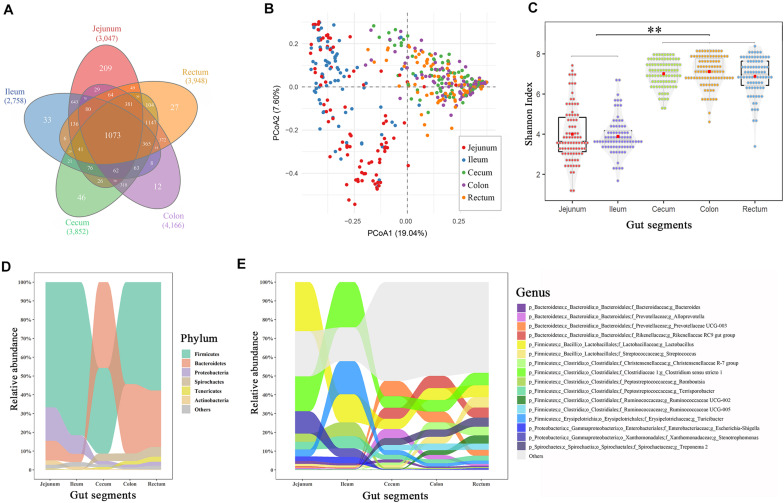
Spatial variations of the microbial composition and diversity across the intestinal tract. **(A)** Venn plot of the OTU numbers in each gut segment. **(B)** Principal coordinate analysis plot based on Bray–Curtis dissimilarities. **(C)** α-Diversity comparison based on the Shannon diversity index, with ANOVA to determine significant differences (^∗∗^*p* < 0.01). **(D)** Relative abundances in the dominant phyla across the intestinal tract. **(E)** Relative abundances of dominant genera across the intestinal tract. Only the genera with an abundance of > 1.0% in any segment are plotted.

The 16S rRNA sequencing generated 21,378,778 clean sequences from 455 samples, with an average of 47,006 reads (Supplementary Table 1). An average of 3,554 OTUs (range 2,758–4,766) were clustered with a 100% sequence identity for each gut segment, with 1,077 OTUs present in all gut segments (Figure 1A). All OTUs were subsequently classified into 30 phyla, 56 classes, 120 orders, 204 families, 492 genera, and 262 species (Supplementary Table 2).

The principal coordinate analysis showed that the taxonomic compositions in the large intestinal segments (cecum, colon, and rectum) were distinctly separated from those of the small intestinal segments (jejunum and ileum; Figure 1B). Additionally, the distal intestines showed a higher α-diversity in the microbiota than the proximal intestines (*p* < 0.01, Figure 1C). Firmicutes, Bacteroidetes, Proteobacteria, Spirochaetes, Tenericutes, and Actinobacteria were the most abundant phyla in the swine guts (∼97%), whereas appreciable differences were observed in the proximal and distal gut segments (Figure 1D). For example, Bacteroidetes accounted for 8–15% of the total microbiota in the jejunum and ileum and 30–45% in the cecum, colon, and rectum. Firmicutes and Proteobacteria displayed the opposite result to that of Bacteroidetes. *Lactobacillus* and *Clostridium* were the two most abundant genera in the jejunum (26.1 and 18.6%, respectively) and ileum (14.9 and 24.1%, respectively), whereas these two genera decreased to 5–6% in the large intestinal segments (Figure 1E). Correspondingly, *Rikenellaceae* and *Prevotellaceae*, which were relatively highly abundant in the cecum, colon, and rectum (∼6%), were nearly undetected in the small intestine (∼0.3%).

**FIGURE 2:**
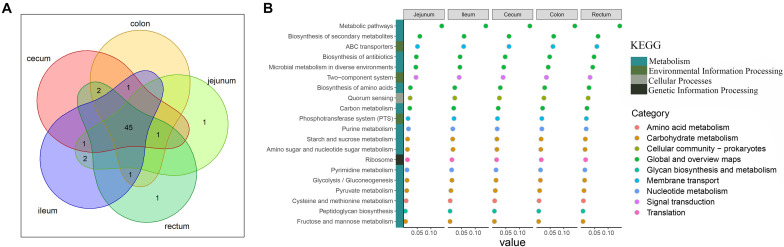
Description of functional capacities of the gut microbiota in each gut segment. **(A)** Overlap of the top 50 predictions across the five gut segments. **(B)** Description of the top 20 functional classifications. *y*-axis lists the functional classifications. *x*-axis shows the sum abundance of microbes in the corresponding function classification. Colored squares indicate different Kyoto Encyclopedia of Genes and Genomes pathways; colored circles indicate the subclassifications in the Kyoto Encyclopedia of Genes and Genomes pathway of metabolism.

Analysis of the functional prediction of the intestinal microbial community showed that the first 50 pathways enriched in each intestinal segment were almost identical (45/50; Figure 2A). Figure 2B shows the top 20 pathways in which the microbes were involved. The major pathways were related to the metabolism of crucial molecules, including amino acids, carbon, purine, starch, sucrose, amino sugar, pyrimidine, pyruvate, cysteine, methionine, fructose, and mannose. The remaining pathways were mainly related to environmental information processing such as ABC transporters, two-component signal transduction systems, and the phosphotransferase system.

### Heritability and Microbiability of Growth and Fat-Related Traits

**FIGURE 3:**
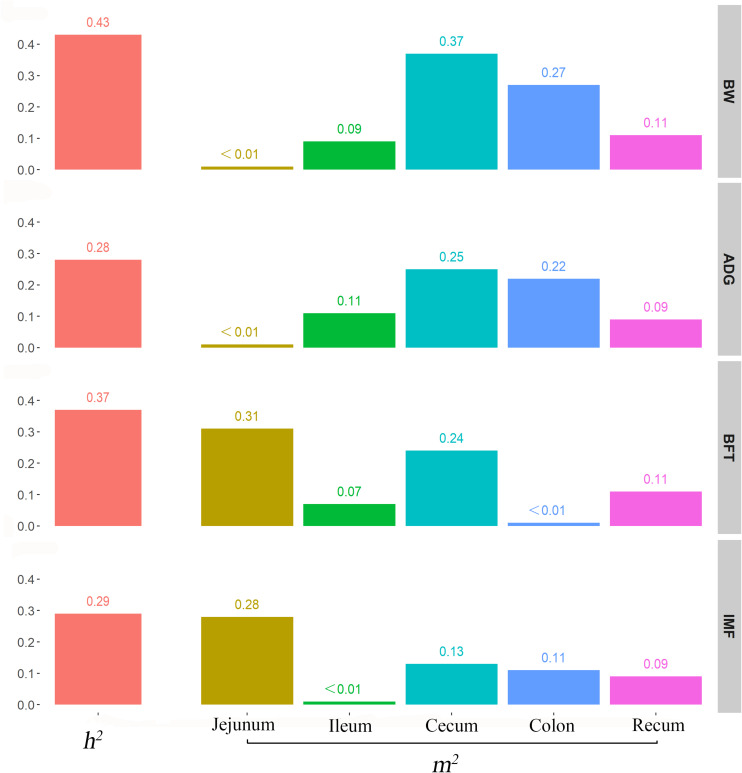
Contribution of host genetics and gut microbiota to target traits. From top to bottom, the figures describe the heritability and microbiability of body weight, average daily gain, backfat thickness, and intramuscular fatness.

We used the traditional indicator, heritability (*h*^2^), to evaluate the degree of genetic variants in determining the target traits and used the newly proposed parameter, microbiability, to dissect the microbiota contribution in each gut segment to the same trait. The relatively high h^2^ values for BW (0.43), ADG (0.28), BFT (0.37), and IMF (0.29) suggested that host genetics play crucial roles in determining pig growth and fat accumulation (Figure 3). The microbiability (*m*^2^) of each gut segment for the different traits varied significantly. The m^2^ values of the BW and ADG estimated for the cecum (∼0.35) and colon (∼0.25) were higher than those estimated for the other segments (range, < 0.01–0.11). For BFT and IMF, m^2^ values were higher for the jejunum (∼0.30) and cecum (∼0.19) than those for the other segments (∼0 zero for the ileum, ∼0.05 for the colon, and ∼0.10 for the rectum) (Figure 3). These results indicate that the colonic and cecal microbiotas play extensive roles in pig growth, and the microbiotas in the jejunum and cecum largely affect fat deposition. To validate the reliability of estimated m^2^, the permutation test was performed for the relatively high m^2^ by randomly reordering the phenotypes for 1,000 times. The results showed that the real m^2^ (0.13–0.37) values were significantly higher than the simulated ones [m^2^ ranged from 0.02 to 0.04, false discovery rate (FDR) < 0.05, Supplementary Figure 1]. Then, the correlation between the genomic relationship matrix and MRM was calculated by the Mantel test; the values varied from −0.0071 to 0.0062 for different gut segments, indicating the relative independence between heritability and microbiability (Supplementary Table 3). We then analyzed whether host genetics can affect microbiota diversity. The α-diversity parameters of the Shannon index, Faith’s phylogenetic diversity, and observed OTUs were used as phenotypes to estimate the SNP-based heritability (Table 2). Host genetics partially determined the microbiota diversities in the colon, cecum, and jejunum, however, the ileum and rectum were minimally affected.

**TABLE 2:** SNP-based heritability of α-diversity indexes for each gut segment.

α-diversity	Heritability				
	Jejunum	Ileum	Cecum	Colon	Rectum
Shannon	0.26	0.14	0.31	0.43	0.13
Observed OTUs	0.21	0.04	0.37	0.39	0.09
Faith PD	0.23	0.08	0.38	0.37	0.21

### Differences in Phyla and Genera Between Divergent Trait Groups

The gut microbiota is involved in pig growth and fat deposition, especially regarding the effects of the cecum and colon on BW and ADG and of the jejunum and cecum on BFT and IMF. Microbial differences at the phylum level were first compared between the highest and lowest 20% of the pigs (high and low groups) according to each trait. Gut segments with higher microbiabilities for a certain trait tended to have larger microbial differences between divergent groups for the same trait; thus, more differences were observed in the cecum, colon, and jejunum, whereas almost no differences were seen in the ileum and rectum (Supplementary Table 4). For the BW and ADG divergent groups, more *Bacteroidetes* (52.6 vs. 44.9%) and fewer *Firmicutes* (40.7 vs. 46.2%) were found in the ceca of the highest 20% pigs than in the lowest 20%, and for the colon, the values were 40.3 vs. 33.0% and 50.5 vs. 56.6%, respectively. For the BFT and IMF divergent groups, the ceca displayed the same trends for *Bacteroidetes* (49 vs. 43%) and *Firmicutes* (43.7 vs. 47.5%), whereas for the jejunum, *Firmicutes* (68.3 vs. 58.3%) and *Proteobacteria* (16 vs. 34.6%) differed, but *Bacteroidetes* did not.

**FIGURE 4:**
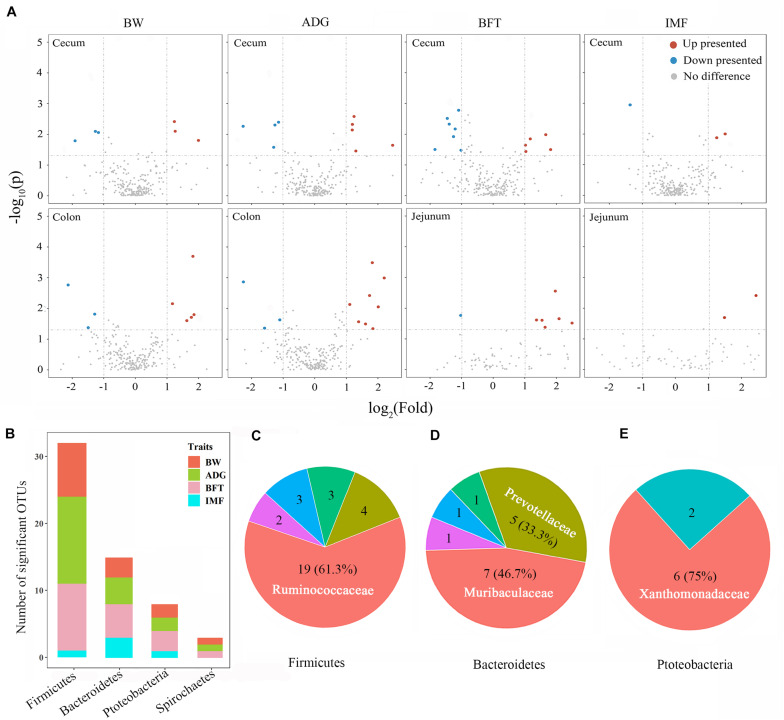
Microbial detections via Wilcoxon rank-sum tests between divergent groups and their distributions. **(A)** Volcano plots for differentially presented microbes in the cecum, colon, and jejunum for BW, ADG, BFT, and IMF. Red points represent upregulated microbes with a log2 (fold change) > 1 and *p* < 0.05; blue points represent downregulated microbes with a log2 (fold change) < -1 and p_*adj*_ < 0.05; gray points represent microbes with no significant difference. Fold change = normalized microbial abundance in the highest 20% group/lowest 20% group. **(B)** Distributions of significant microbes on phyla and traits. **(C–E)** Major families to which the significant microbes belong within the phyla of Firmicutes, Bacteroidetes, and Proteobacteria.

Wilcoxon rank-sum tests were performed for the divergent groups to detect the associated individual microbes, and only the gut segments with relatively high microbiability were further screened. Fifty-eight microbes differed significantly among the gut segments between the divergent groups for the four target traits (Figure 4A and Supplementary Table 5). These microbes belonged to the phyla *Firmicutes* (55.2%), *Bacteroidetes* (25.9%), *Proteobacteria* (13.8%), and *Spirochaetes* (5.2%; Figure 4B). For *Firmicutes*, the family *Ruminococcaceae* constituted the largest proportion (61.3%, Figure 4C) and was higher in the ceca and colons of the high group for BW, ADG, and BFT (2.1–4.5-fold than the low group). Two families, *Muribaculaceae* and *Prevotellaceae*, accounted for 80% of all Bacteroidetes and were both upregulated in pigs in the high group by 2.3–4.2-fold (Figure 4D). Conversely, for *Proteobacteria* (*Xanthomonadaceae* constituted 75%, Figure 4E) and *Spirochaetes* (only from the *Spirochaetaceae* family), all microbes were upregulated in pigs of the low group (2.1–4.7-fold than the high group; Supplementary Table 5).

### Correlation Analysis of the Screened Microbes and Target Traits

**FIGURE 5:**
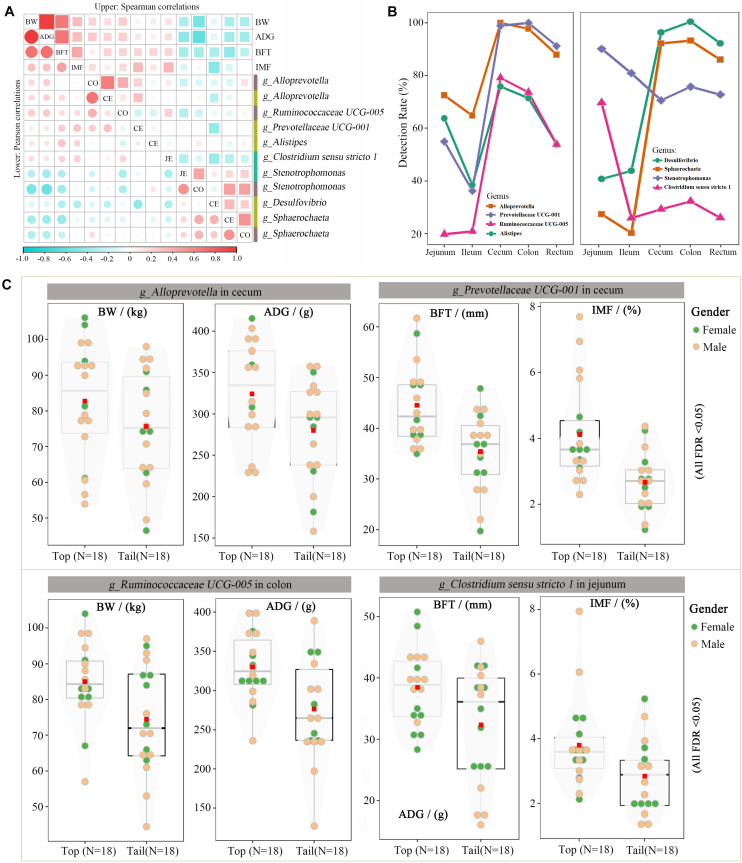
Candidate microorganisms for growth and fat-related traits. **(A)** Correlation coefficients for the BW, ADG, BFT, IMF, and eight microbial genera. Upper and lower diagonals indicate the Pearson and Spearman correlations, respectively. Diagonal shows the traits and gut segments (JE: jejunum, CE: cecum, and CO: colon). **(B)** Detection rate of these seven microbial genera across the intestinal tract. **(C)** Differences in BW, ADG, BFT, and IMF between the top and bottom abundances of *Ruminococcaceae* UCG 005, *Prevotellaceae* UCG 001, *Stenotrophomonas*, and *Desulfovibrio*.

Many of these 58 microbes overlapped, owing to their associations with multiple traits, such as the cecal genus of *Stenotrophomonas*, which was simultaneously associated with BW, ADG, and BFT. After removing the overlapped taxa, 39 unique genus-level microbes were screened. Microbes with detection rates > 65% (25 microbes) were subsequently used to calculate Pearson’s and Spearman’s correlation coefficients with the target traits. Each microbe had eight correlation coefficients with four traits, and only microbes with at least one significant *p*-value were retained (11 microbes, Figure 5A). *Alloprevotella* (colon and cecum) and *Ruminococcaceae* UCG-005 (colon) were positively correlated with BW and ADG, and *Prevotellaceae* UCG-001 (cecum), *Alistipes* (cecum), and *Clostridium sensu stricto 1* were positively correlated with BFT and IMF. The remaining genera were negatively correlated with the target traits. Specifically, *Stenotrophomonas* (jejunum and colon) and *Sphaerochaeta* (cecum and colon) were negatively correlated with BW, ADG, and BFT, and *Desulfovibrio* (cecum) was negatively correlated with BFT and IMF. Notably, *Alloprevotella*, *Stenotrophomonas*, and *Sphaerochaeta* were simultaneously correlated with the target traits in two separate gut segments and extremely highly positively correlated (*r* = 0.8–0.9) within each genus pair. The spatial distributions of these eight candidate microbes were characterized across the intestinal tract (Figure 5B), and the cecum and colon had the highest detection rates, whereas the ileum had the lowest, thus indicating the crucial roles of the microbiota in the cecum and colon.

### Cross-Validation of Candidate Microbes for Growth and Fat-Related Traits

The divergent Wilcoxon rank-sum test and correlation analysis revealed 11 associations corresponding to eight microbial genera. The phenotypic differences in target traits were compared between the highest and lowest 20% of the pigs (the top and bottom groups) with the candidate microbes. Figure 5C illustrates the effects of *Alloprevotella* (cecum), *Prevotellaceae* UCG-001 (cecum), *Ruminococcaceae* UCG-005 (colon), and *Clostridium sensu stricto 1* (jejunum) on the target traits. These four genera had the largest positive/negative correlation coefficients with at least one target trait. For example, *Ruminococcaceae* UCG-005 in the colon was highly positively correlated with BW and ADG; hence, the BW and ADG were significantly higher in the top *Ruminococcaceae* UCG-005 group than those in the bottom group (BW was 85.0 vs. 74.4 kg, FDR < 0.05; ADG was 330.2 vs. 276.5 g, FDR < 0.05).

## Discussion

The gut microbiota plays an essential role in animal health (Lee and Hase, 2014). The microbial communities are diverse and complex in the pig gastrointestinal tracts and are involved in gut-associated immune activity and assist the host in digesting feed nutrients. Genetic factors are the most important determinants for desirable traits in farm animals, whereas the gut microbiota is an additional factor because of its diverse interactions with the host (Lu et al., 2018).

Previous microbiota association studies focused mainly on the fecal microbiota or microbes in a specific gut segment such as the cecum. However, feces derived from the whole intestinal tract can qualitatively but not quantitatively represent the gut microbiota abundance of each segment (Yan et al., 2019). Interactions between the host and its gut microbiota occur between individual microbes and gut epithelial cells via metabolite-based signal transductions. Hence, the microbiotas of different gut segments might influence different traits. The current study also validated the microbial differences across the whole intestinal tract, in which the major microbiota phyla, including Firmicutes, Bacteroidetes, Proteobacteria, and Spirochaetes, varied widely from the proximate to the distal gut. Differences in oxygen availability (Espey, 2013), pH (Duncan et al., 2009), and nutrient variety (Rowland et al., 2018) in each segment contribute to the spatial heterogeneity along the intestinal tract. Consequently, in the current study, the microbial diversity in each location differed, and the proximal intestinal tract was less diverse than the distal part for favorable conditions for fermenting resistant polysaccharides, cellulose, and starch.

In the current study, host genetics played major roles in growth and fat-related traits. BW and ADG are the main traits for evaluating swine growth performance. Fat serves as the primary energy source, but for human consumption, the subcutaneous fat is considered excessive fat accumulation. Conversely, the intramuscular fat is considered a determinant of meat flavor and favored by consumers. Our estimations of the SNP-based h^2^ values for BW, ADG, BFT, and IMF were moderate to high (0.43, 0.28, 0.37, and 0.29, respectively) and were comparable with those of previous studies (Lutaaya et al., 2001; Casellas et al., 2010), indicating the considerable effects of host genetics. In the classic theory of quantitative genetics, the residual effect for complex traits is the environment, which is unstable and difficult to change and use. However, with the appearance and prevalence of the gut microbiome, animals are considered superorganisms, or symbionts, comprising animals and the microbes they contain. Microorganisms enable animals to have biological functions that their own genomes cannot achieve. Additionally, gut microorganisms interact with host genetics; thus, they adapt to genetic changes in the host. Hence, the influence of the gut microbiota on host complex traits can be considered a stable effect that was originally embedded in the environmental effect. “Microbiability” can be used to quantify this effect of gut microbes and is defined as the proportion of phenotypic variances that can be inferred from the gut microbiota (Difford et al., 2018). Larger m^2^ values mean a greater effect of the gut microbiota on target traits and deserve more elaborate analysis. The m^2^ estimates in the current study suggest that the jejunal, cecal, and colonic microbial communities are more important predictors of growth and fat-related traits, contributing to 22–37% of the BW and ADG and 13–31% of the BFT and IMF. The cecum and colon have relatively high m^2^ because they are part of the distal gastrointestinal tract, which are the major anaerobic sites for fermentation and SCFA production (Butine and Leedle, 1989); SCFAs can be absorbed and serve as energy sources for the host. For jejunal, it is the major part of the production of secondary bile acid from preliminary bile acid, relying on their resided microbials (Tripathi et al., 2018). The bile acid and its interaction with gut microbials are crucial for fat metabolism, which might lead to high m^2^ of jejunal microbiota for fat-related traits. Our results showed that the relationships between host genetic kinship and microbial similarities were very weak, which were consistent with the studies in humans (Rothschild et al., 2018), chicken (Wen et al., 2019), and horse (Massacci et al., 2019). In contrast, some other studies found significant but weak correlations ranging from 0.14 to 0.19 (Blekhman et al., 2015; Suzuki et al., 2019). Population structure, genetic relatedness, food intake, and environmental factors may affect the differences among varied studies. However, these observations together indicated that the host genome had a relatively weak influence on the gut microbiome as a whole.

The substantial effect of the microbiotas in the cecum, colon, and jejunum on growth and fat-related traits led us to detect the individual microbes that play crucial roles in this linkage. Of the 10 screened microbes, 9 were in the cecum and colon, indicating that the cecum and colon play more important roles than the jejunum. *Alloprevotella* in the cecum and colon and *Ruminococcaceae* UCG-005 in the colon were highly positively correlated with BW and ADG. *Alloprevotella* is an SCFA-producing genus that produces moderate levels of acetic acid and major amounts of succinic acid and has saccharolytic properties (Downes et al., 2013). In the current study, genus *Alloprevotella* positively related to host growth, which was consistent with the findings of Liu et al. (2018), who suggested that a high abundance of *Alloprevotella* in the gut after feeding fucoidan can improve the physical health of mice such as by alleviating dyslipidemia. Previous studies have suggested that *Ruminococcaceae* UCG-005 also benefits the host by protecting against diabetes and elevating intestinal SCFA levels (Andrade et al., 2020). Wang et al. (2018) found that the abundance of *Ruminococcaceae* UCG-005 was significantly reduced in the intestines of diarrheic goat kids compared with those of healthy goat kids. We found that *Prevotellaceae* UCG-001 and *Alistipes* in the cecum were highly positively correlated with BFT and IMF. *Prevotellaceae* UCG-001, a strain of *Prevotella* with enzymes that can degrade cellulose and xylan, is also considered a beneficial bacterium. *Prevotellaceae* UCG-001 is significantly enriched after feeding inulin to mice, thus alleviating glucose and lipid metabolism disorders (Song et al., 2019). In chickens, increased *Prevotellaceae* UCG-001 in the cecum was correlated with improved laying performance and egg quality in hens (Sun et al., 2020). *Alistipes finegoldii* has been relatively comprehensively studied and is involved in lipid metabolism via its bacterial type II fatty acid biosynthesis system for producing saturated fatty acids ≤ 16 carbons (Radka et al., 2019).

*Stenotrophomonas*, *Sphaerochaeta*, and *Desulfovibrio* were negatively correlated with the target traits. *Stenotrophomonas* comprises at least eight environmental species (Ryan et al., 2009). *Stenotrophomonas maltophilia* is involved in beneficial processes in plants but is commonly associated with respiratory infections in humans and is considered an emerging global opportunistic pathogen (Brooke, 2012). In the current study, the high abundance of *Stenotrophomonas* reduced pig growth and fat deposition, indicating its negative role in animal guts. *Sphaerochaeta* includes anaerobic, mesophilic, and slightly halophilic microbes, which use many carbohydrates and grow as biofilms (Pajarillo et al., 2014). Morrison et al. (2020) indicated the roles of *Sphaerochaeta* in equine weight loss. *Desulfovibrio* spp. belong to a group of anaerobic and sulfate-reducing bacteria with more than 30 species (Goldstein et al., 2003). This sulfate-reducing bacterium may produce H_2_S in anaerobic animal guts (St-Pierre and Wright, 2017). H_2_S is deemed an undesirable by-product of anaerobic digestion and is an animal health hazard even at low concentrations. Our results showed that a high abundance of *Desulfovibrio* in pig guts could reduce production performance, possibly because the extra H_2_S production in the cecum reduces the animals’ health.

## Conclusion

In the current study, we performed 16S rRNA gene sequencing on the jejunal, ileal, cecal, colonic, and rectal microbiotas in swine. Owing to the relatively high microbiability, the cecal, colonic, and jejunal microbiotas played more crucial roles in determining growth and fat-related traits than those of the ileum and rectum, and we screened a list of genus-level microbes. Our results provide a more comprehensive understanding of the contribution of the gut microbiota in separate gut segments to pig growth and fat-deposition traits and suggest that target traits may be changed by manipulating gut microbes.

## Data Availability Statement

The original contributions generated for the study are publicly available. This data can be found in NCBI, under accession PRJNA647786.

## Ethics Statement

The animal study was reviewed and approved by the Scientific Ethics Committee of Huazhong Agricultural University. Written informed consent was obtained from the owners for the participation of their animals in this study.

## Author Contributions

ST: sample collection, data analysis, and wrote the manuscript. YX and YM: measurement of pig phenotype and sample collection. XX: sample collection. SZ and JC: conceived and designed the experiments and manuscript revision. All authors contributed to the article and approved the submitted version.

## Conflict of Interest

The authors declare that the research was conducted in the absence of any commercial or financial relationships that could be construed as a potential conflict of interest.

## Supplementary Material

Supplementary Figure 1 Permutation test for microbiability of cecum, colon and jejunum on target traits.

Supplementary Table 1 Descriptive statistics for reads sequenced in each gut segment.

Supplementary Table 2 The identification of phyla, classes, orders, families, genera and species in the current study.

Supplementary Table 3 Correlation between genomic relationship matrix and microbial relationship matrix.

Supplementary Table 4 phyla proportion in each gut segment of divergent groups of ADG, BW, BFT and IMF.

Supplementary Table 5 Description of 58 significantly presented microbes that associated with four target traits.
