# Supplementary figures and images for: Screening of Microbes Associated With Swine Growth and Fat Deposition Traits Across the Intestinal Tract
Source: Front Microbiol. 2020 Oct 16;11:586776. doi: 10.3389/fmicb.2020.586776 (PMC7596661; doi:10.3389/fmicb.2020.586776)

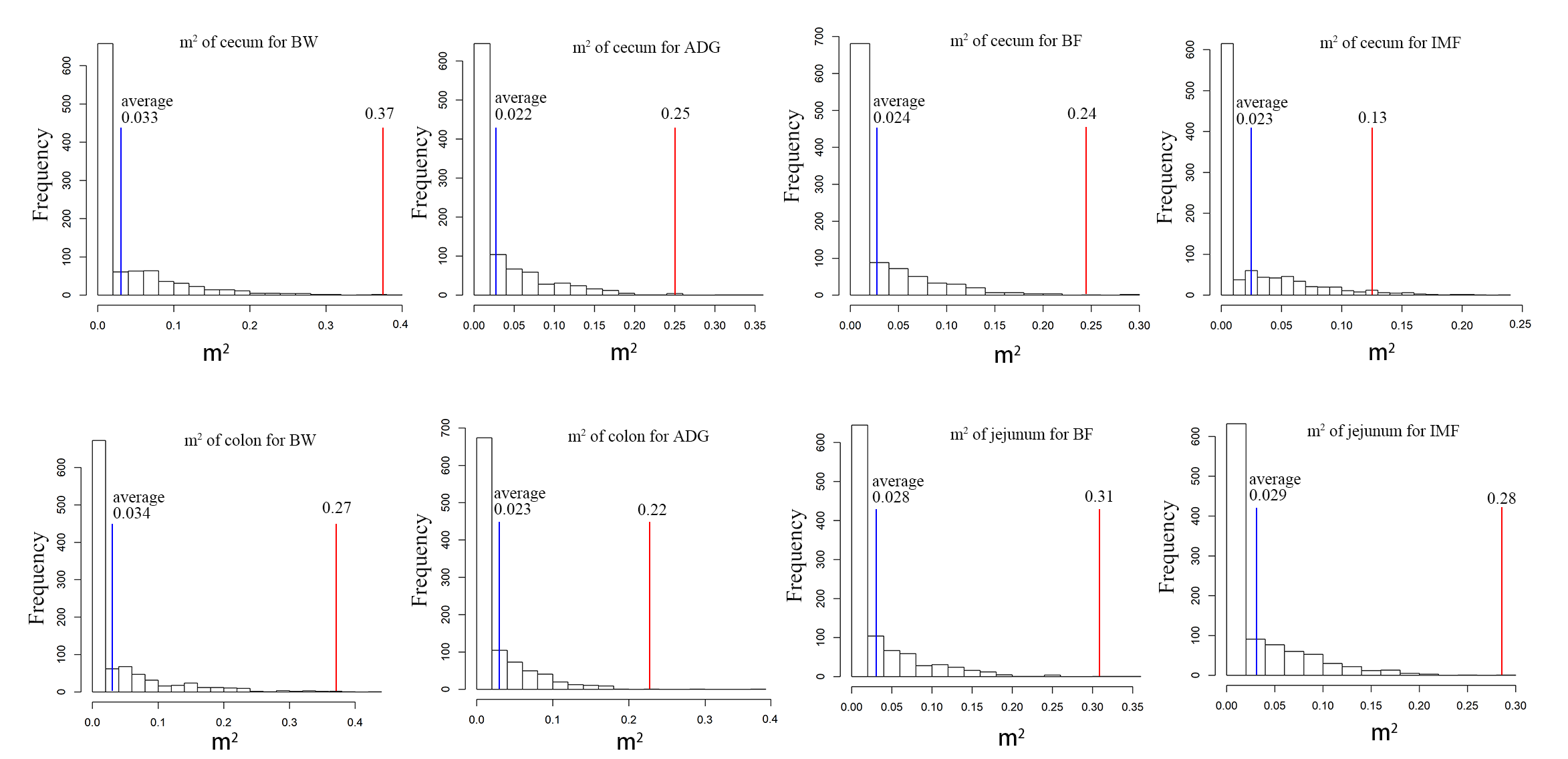

Supplement: Supplementary Figure 1 — Permutation test for microbiability of cecum, colon and jejunum on target traits. [file Image_1.tif]
